# Macrophage adaptation leads to parallel evolution of genetically diverse *Escherichia coli* small‐colony variants with increased fitness in vivo and antibiotic collateral sensitivity

**DOI:** 10.1111/eva.12397

**Published:** 2016-06-30

**Authors:** Ricardo S. Ramiro, Henrique Costa, Isabel Gordo

**Affiliations:** ^1^ Instituto Gulbenkian de CiênciaOeirasPortugal

**Keywords:** antibiotic resistance, collateral sensitivity, experimental evolution, macrophages, mouse gut colonization, small‐colony variants, whole‐genome sequencing

## Abstract

Small‐colony variants (SCVs) are commonly observed in evolution experiments and clinical isolates, being associated with antibiotic resistance and persistent infections. We recently observed the repeated emergence of *Escherichia coli* SCVs during adaptation to the interaction with macrophages. To identify the genetic targets underlying the emergence of this clinically relevant morphotype, we performed whole‐genome sequencing of independently evolved SCV clones. We uncovered novel mutational targets, not previously associated with SCVs (e.g. *cydA*,* pepP*) and observed widespread functional parallelism. All SCV clones had mutations in genes related to the electron‐transport chain. As SCVs emerged during adaptation to macrophages, and often show increased antibiotic resistance, we measured SCV fitness inside macrophages and measured their antibiotic resistance profiles. SCVs had a fitness advantage inside macrophages and showed increased aminoglycoside resistance in vitro, but had collateral sensitivity to other antibiotics (e.g. tetracycline). Importantly, we observed similar results in vivo. SCVs had a fitness advantage upon colonization of the mouse gut, which could be tuned by antibiotic treatment: kanamycin (aminoglycoside) increased SCV fitness, but tetracycline strongly reduced it. Our results highlight the power of using experimental evolution as the basis for identifying the causes and consequences of adaptation during host‐microbe interactions.

## 1 Introduction

Variation in colony morphology is often observed during in vitro bacterial adaptation (Miskinyte et al., 2013; Rainey & Travisano, 1998; Rozen & Lenski, 2000; Traverse, Mayo‐Smith, Poltak, & Cooper, 2013). One of the most commonly observed morphotypes are small‐colony variants (SCVs) (e.g. Miskinyte et al., 2013; Rozen & Lenski, 2000; Traverse et al., 2013). SCVs emerge in response to multiple selective pressures, including adaptation to: single (Rozen & Lenski, 2000) or multiple carbon sources (Friesen, Saxer, Travisano, & Doebeli, 2004), biofilm conditions (Penterman et al., 2014; Traverse et al., 2013), the mouse gut (Lee et al., 2010), antibiotics (Musher, Baughn, Templeton, & Minuth, 1977; Yegian, Gallo, & Toll, 1959), antimicrobial peptides (Pranting & Andersson, 2010) or the intracellular environment of nonprofessional phagocytes (Cano, Pucciarelli, Martínez‐Moya, Casadesús, & García‐del Portillo, 2003; Tuchscherr et al., 2011; Vesga et al., 1996). As could be expected, from such a diverse set of selective pressures, SCVs also present a diverse genetic basis (Cooper, Staples, Traverse, & Ellis, 2014; Herron & Doebeli, 2013; Pranting & Andersson, 2010; Proctor et al., 2006). The most common mutations are in genes related to the electron‐transport chain (ETC), which are known to lead to the SCV phenotype in many species, both Gram‐negative and Gram‐positive (Cano et al., 2003; Dean, Olsen, Long, Rosato, & Musser, 2014; Kastbjerg, Hein‐Kristensen, & Gram, 2014; Roggenkamp et al., 1998). Importantly, SCVs have been widely associated with antibiotic resistance, and for some of these species (e.g. *S. aureus, E. coli*,* Pseudomonas aeruginosa*), SCVs have been associated with chronic infections, such as infections of the lungs of cystic‐fibrosis patients, urinary tract or prosthesis‐associated infections (Häussler, Tümmler, Weißbrodt, Rohde, & Steinmetz, 1999; Proctor, van Langevelde, Kristjansson, Maslow, & Arbeit, 1995; Proctor et al., 2006; Roggenkamp et al., 1998; Rusthoven, Davies, & Lerner, 1979). In these natural infections, disentangling which selective pressures lead to particular phenotypes is often difficult. Interestingly, some of these SCVs show similar genetic/phenotypic bases to those which arise during evolution experiments (McDonald, Gehrig, Meintjes, Zhang, & Rainey, 2009; Proctor et al., 1995; Smith et al., 2006; Vesga et al., 1996), suggesting that experimental evolution may be useful to identify important selective pressures in vivo.

A recent aim in evolutionary biology is to understand whether evolution experiments can help understanding natural variation. A way to do this is to incorporate the selective pressures which are thought to be important in nature into evolution experiments (Bailey & Bataillon, 2016). In the context of infections and at the commensal–pathogen transition, a major selective pressure is the innate immune system, with macrophages (MΦs) being key in the response to microbial infections (Wynn, Chawla, & Pollard, 2013). Due to this role of MΦs, several studies have recently used experimental adaptation to understand how yeast or bacteria evolve under MΦ attack (Azevedo et al., 2016; Ensminger, Yassin, Miron, & Isberg, 2012; Miskinyte et al., 2013; Vázquez et al., 2014; Wartenberg et al., 2014). Recently, we used this approach to study the adaptation of a commensal *E. coli* to the interaction with MΦs (Miskinyte et al., 2013). In this experiment, replicate bacterial populations were allowed to evolve for 30 days in the presence of MΦs, while inhabiting both intracellular and extracellular environments (see Appendix S1 for summary of this experiment). Rapid and repeated emergence of SCVs occurred in independent bacterial populations, indicating that SCVs are an adaptive trait in the interaction with this key immune cell (Dettman et al., 2012; Elena & Lenski, 2003).

Here, we used whole‐genome sequencing (WGS) of multiple clones to understand what is the genetic basis of SCVs and if the phenotypic parallelism is mediated by genetic parallelism. Our analysis shows evidence of parallelism from the gene to the pathway level and indicates widespread functional parallelism: all SCVs had mutations in genes related to the ETC, a common observation in clinically isolated SCVs (Proctor et al., 1995; Roggenkamp et al., 1998). Importantly, we identified several mutational targets that had not previously been associated with SCVs, including *nuoG*,* hemY* or *ubiG*. As is often observed for ETC‐deficient SCVs (Proctor et al., 2006), we show that these SCV clones generally have a high reversion rate, are hemin auxothrophs, and generally reach a lower carrying capacity than the ancestral strain, both in poor and rich media. As SCVs emerged in the presence of MΦs, and such morphotypes have previously been shown to have increased antibiotic resistance, we tested for SCV fitness inside MΦs and for antibiotic resistance in vitro and upon colonization of the mouse gut. Our results show that SCVs can have an intracellular advantage inside MΦs, and that this morphotype has a pattern of antagonistic pleiotropy for antibiotic resistance in vitro: SCVs were more resistant to aminoglycosides but more sensitive to tetracycline, nalidixic acid and ciprofloxacin (quinolones), as well as cefotaxime (a β‐lactam). Notably, upon colonization of streptomycin‐treated mice, SCVs demonstrated a fitness advantage that could be increased upon kanamycin treatment (an aminoglycoside) or decreased by nalidixic acid or tetracycline (as predicted by in vitro results), with tetracycline treatment strongly reducing SCV frequency in the mouse gut. Therefore, our results show that *E. coli* SCVs that emerge in the presence of MΦs are caused by mutations in a common pathway and have important pleiotropic consequences for host‐microbe interactions.

## 2 Material and Methods

### 2.1 Isolation of SCV clones, reference bacterial strains and MΦs

We isolated SCV clones from a previous experiment, in which *E. coli* was adapted to MΦs (see Appendix S1). These SCV clones were evolved from an ancestral strain of commensal *E. coli* (MC4100‐CFP), which is streptomycin resistant (RpsL^K43R^) and constitutively expresses cyan‐fluorescent protein (CFP; galK::CFP, Amp^R^). SCVs were identified in five of six independently evolving populations (Miskinyte et al., 2013; exception was population M4). Stocks of two randomly selected SCV clones (per population) were made and kept at −80°C. For population M4, two clones were also isolated. These clones produce colonies of similar size to the ancestral strain (see Fig. S1 for colony sizes) and were used as positive controls (i.e. clones that evolved with MΦs, but do not display a SCV phenotype) for the experiments below.

For competition assays, strain MC4100‐YFP was used as reference strain. This differs from MC4100‐CFP only in the expressed fluorescent protein. For the catalase activity test, a Δ*rpoS* strain was used as a negative control (Al Mamun et al., 2012). The RAW 264.7 murine MΦ cell line was used to test for SCV intracellular survival (see Appendix S1 for MΦ‐culture conditions).

### 2.2 Whole‐genome sequencing and mutation prediction

Bacterial clones were grown in 10 ml RPMI at 37°C with 5% CO_2_. DNA was extracted using the procedure described by Wilson (2001). DNA library construction and sequencing was performed by the IGC genomics facility. Each clone was paired‐end sequenced on an Illumina MiSeq Benchtop‐Sequencer. Standard procedures produced data sets of Illumina paired‐end 250 bp read pairs, with mean coverage of 55× (32×–113× per clone). The genome sequencing data have been deposited in the NCBI Sequence Read Archive www.ncbi.nlm.nih.gov/sra (accession no.: SRP074322). *breseq* v0.20 was used to map reads and identify mutations, using the *E. coli* BW2952 genome as reference (NCBI accession: NC_012759.1; Deatherage & Barrick, 2014). Default settings were used, except for eliminating mutation predictions occurring in homopolymers of length >3. Mutations were determined by comparison with the ancestral (MC4100‐CFP; previously sequenced for (Miskinyte et al., 2013). All predicted polymorphisms were manually inspected using IGV. Functional annotation clustering was performed using DAVID (Huang, Sherman, & Lempicki, 2008, 2009), with standard settings.

### 2.3 Phenotypic traits of SCVs

Whole‐genome sequencing revealed that SCVs had mutations in genes associated with the ETC. These SCVs often show the following phenotypes: (i) high reversion frequency to large colony phenotype; (ii) reduced catalase activity; (iii) slow growth in vitro; and (iv) auxotrophy for compounds involved in the ETC (Proctor et al., 2006). To test whether our SCVs had these phenotypes, we (i) measured reversion frequency (to large colony phenotype) and catalase activity as described in Miskinyte et al. (2013) and Roggenkamp et al. (1998) (see Appendix S1); (ii) estimated the relative growth rate and relative carrying capacity of each SCV (relative to the ancestral) in rich (RPMI) and poor media (M9 minimal media with 0.4% glucose); and (iii) tested for auxotrophy to hemin and aminolevulinic acid (5‐ALA) by estimating the lag phase, maximum growth rate and carrying capacity in the presence of these compounds relative to that obtained in its absence. Carrying capacity is defined as the maximum number of individuals (measured by OD) that can be supported by the environment (see Appendix S1 for further description of these assays).

### 2.4 Bacterial survival inside MΦs

Three independently evolved clones carrying distinct mutations (28 and 41 as SCVs and 31 as a positive control; see Table 1) were competed against the YFP ancestral strain in the intracellular environment of MΦs. MΦs were seeded and activated, following a modification of Miskinyte & Gordo (2014) (see Appendix S1). On infection day, media were removed from each well and replaced by 1 ml RPMI with 10^7^ bacteria, a ~50:50 mixture of the YFP ancestral and one of the evolved (CFP) clones (three wells per clone‐time point combination). Cultures were spun down (200 *g*, 5 min.) and plates incubated for 0.5 hr (37°C, 5% CO_2_). After this period, media were removed from all wells and cells washed twice with RPMI to remove extracellular bacteria. RPMI with gentamicin was added to two sets of wells (100 μg/ml), to kill remaining extracellular bacteria and plates incubated for another 0.5 hr (i.e. total incubation time = 1 hr) or 5.5 hr (i.e. total = 6 hr). About 1 ml PBS was added to the remaining set of wells, and MΦs were detached and the contents of each well centrifuged to lyse MΦs (9400 *g*, 5 min). The pellet was then resuspended in 1 ml PBS. This allowed estimation of the number of bacteria that had been phagocytized by MΦs at 0.5 hr. At the 1 and 6 hr timepoints, MΦs were washed, detached and centrifuged, as for the 0.5 hr timepoint. All timepoints were plated, and the number of YFP (ancestral) and CFP (SCVs) bacteria was used to calculate the competitive index (CI) as

(1) $$\text{CI}=\log_{10}\left(\frac{{\text{CFP}}_{xh}}{{\text{YFP}}_{xh}}/\frac{{\text{CFP}}_{0.5\;\mathrm{hr}}}{{\text{YFP}}_{0.5\;\mathrm{hr}}}\right),$$

where *xh* represents 1‐ or 6‐hr incubation, CFP (or YFP) represents the colony‐forming units (cfu) at a particular time point. Six independent replicates were performed, per clone. We used gentamicin, a standard antibiotic for bacterial intracellular survival assays, due to its reduced ability to enter cells and kill intracellular bacteria (Cano et al., 2003; Carryn, Van Bambeke, Mingeot‐Leclercq, & Tulkens, 2002; Nguyen et al., 2009). To ensure that the observed fitness effects are due to environmental conditions inside MΦs and not because gentamicin increases SCV relative fitness, we carried out in vitro competitions in the absence of MΦs. These mimic the competitions with MΦs: 50:50 mixtures (10^7^ bacteria) of each SCV clone and the YFP ancestral were inoculated into RPMI (1 ml), with gentamicin at 100 μg/ml, at pH 5 (mimicking the MΦ intracellular environment) or 7.4 (standard for RPMI). Cultures were incubated for 6 hr and then plated. In three independent experiments (per SCV clone), SCV or ancestral colonies were only rarely observed, indicating that most bacteria (both SCVs and ancestral) cannot survive (Table S1). Moreover, we also carried competitions between isogenic CFP and YFP ancestral (Fig. S2) and observe that the CI was not significantly different from 0 at both 1 hr (*t*_4_ = 0.43, *p* = .69) and 6 hr (*t*_4_ = 1.47, *p* = .22), indicating that the fluorescent markers are neutral in this environment.

**Table 1:** Mutations identified in *E. coli* clones evolved in the presence of MΦs. Deletions (Δ), transposition of insertion sequences (IS) or small nucleotide insertions (+) are shown at the specific position of each mutation, within the gene. For SNPs, the particular nucleotide and corresponding amino acid replacement change is indicated. The deletion in clone 1 includes both intergenic and coding regions, starting at an intergenic position and ending within the cydA coding region. Genomic positions are indicated relative to the GenBank reference: NC_012759.1

Population	Day	Clone no.	Position	Gene	Mutation	Annotation	Product
M1	8	1	673,439	*mngB/[cydA]*	Intergenic (+844/−3) – coding (5/1,569 nt)	Δ6 bp	*cydA*: cytochrome bd‐I terminal oxidase, subunit I; *mngB*: alpha‐mannosidase
M1	8	61	174,223	*hemL*	A→C	L220R (CTG→CGG)	Glutamate‐1‐semialdehyde aminotransferase
			3,077,939	*yqiJ*	Coding (562/630 nt)	IS5	Predicted inner membrane protein
			4,205,688	*yjbS*	Coding (193/204 nt)	IS1	Hypothetical protein
M2	4	11	174,688	*hemL*	C→T	G65D (GGC→GAC)	Glutamate‐1‐semialdehyde aminotransferase
M2	4	19	905,376	*ycbU*	Coding (297/543 nt)	IS2	Predicted fimbrial‐like adhesin protein
			2,223,615	*ubiG*	Coding (222–231/723 nt)	Δ10 bp	Bifunctional 3‐demethylubiquinone‐8 3‐O‐methyltransferase and 2‐octaprenyl‐6‐hydroxyphenol methylase
M3	5	26	174,223	*hemL*	A→C	L220R (CTG→CGG)	Glutamate‐1‐semialdehyde aminotransferase
			3,077,939	*yqiJ*	Coding (562/630 nt)	IS5	Predicted inner membrane protein
M3	5	28	174,223	*hemL*	A→C	L220R (CTG→CGG)	Glutamate‐1‐semialdehyde aminotransferase
			3,077,939	*yqiJ*	Coding (562/630 nt)	IS5	Predicted inner membrane protein
			4,205,688	*yjbS*	Coding (193/204 nt)	IS1	Hypothetical protein
M5	8	41	2,939,858	*pepP*	Coding (153/1,326 nt)	IS1	Proline aminopeptidase P II
M5	8	48	1,069,386	*ndh*	C→T	H52Y (CAC→TAC)	NADH:ubiquinone oxidoreductase II
			2,283,177	*nuoG*	G→C	Y272* (TAC→TAG)	NADH:ubiquinone oxidoreductase I, chain G
M6	4	51	2,927,004	*ygfZ*	Coding (522/981 nt)	+GC	Folate‐binding protein
			3,874,865	*hemY*	T→A	D237V (GAT→GTT)	Predicted protoheme IX synthesis protein
M6	4	52	2,927,004	*ygfZ*	Coding (522/981 nt)	+GC	Folate‐binding protein
			3,874,865	*hemY*	T→A	D237V (GAT→GTT)	Predicted protoheme IX synthesis protein
M4	5	31	4,324,488	*mscM*	C→G	W547C (TGG→TGC)	Mechanosensitive channel of miniconductance, monomer
M4	5	40	4,324,488	*mscM*	C→G	W547C (TGG→TGC)	Mechanosensitive channel of miniconductance, monomer

### 2.5 In vitro antibiotic resistance assays

Antibiotic sensitivity was measured using disc diffusion assays (on LB‐agar plates) for the following antibiotics: gentamicin (10 μg), kanamycin (30 μg), amikacin (30 μg), tobramycin (10 μg), netilmicin (10 μg), cefotaxime (30 μg), ciprofloxacin (5 μg), nalidixic acid (30 μg) and tetracycline (30 μg). The inhibition halo was measured 48 hr postincubation, at 37°C (3–8 independent replicates per clone‐antibiotic combination).

### 2.6 In vivo competition assays in the mouse gut

To test the competitive ability of SCVs in vivo, we used the streptomycin‐treated mouse model of *E. coli* colonization (Conway, Krogfelt, & Cohen, 2004). This model enables colonization by *E. coli*, while maintaining a diverse microbiota (Barroso‐Batista, Demengeot, & Gordo, 2015). Briefly, 8‐week‐old C57BL/6 female mice raised in specific pathogen‐free conditions (ad libitum food), were given autoclaved water with streptomycin (5 g/L) for 7 days before and after colonization (total = 14 days). Mice were caged in pairs and gavaged with a suspension of 10^8^ bacteria (in 100 μl PBS; as in Barroso‐Batista et al. (2014)), consisting of a mix of SCV‐CFP and ancestral YFP (ancestral and SCV carry the streptomycin‐resistance allele RpsL^K43R^; Trindade et al., 2009). The SCV population contained a mixture of equal numbers of the following genetically distinct clones: 1, 19, 26, 28, 41, 48 and 51, which cover all the mutations observed in our experiment (see Table 1). Groups of four mice (caged in pairs) were used for each experimental group. Specifically, one group did not receive any additional treatment (‘no treatment’ in Fig. 3) and the other three groups were gavaged (between days 1 and 6) with the following antibiotics: kanamycin (1 mg), tetracycline (0.5 mg) or nalidixic acid (2 mg). Doses were based on per kg doses administered to human adults.

Mice in different cages were inoculated with independently grown bacteria and on different days. After *E. coli* gavage, faecal samples were obtained daily, homogenized and plated (LB‐agar) for estimating CFP (SCV or revertant) and YFP (ancestral) cfu. As a control, four mice were also gavaged with a mix of isogenic CFP/YFP ancestral. For these mice, the YFP/CFP ratio did not significantly change over time ($\chi_{1}^{2}$ *= *0.82, *p* = .36; Fig. S3), indicating that the fluorescent markers are neutral in this environment. Moreover, to ensure that the observed fitness effects are due to the environmental conditions in the gut and not because streptomycin increases the relative fitness of SCVs, in vitro competitions were carried, mimicking the in vivo competitions. This consisted of inoculating a 50:50 mixture of the SCV population and the YFP ancestral into RPMI at 100 μg/ml or 500 μg/ml of streptomycin (*n* = 3 replicates per concentration). Twenty‐four hours postincubation, the frequency of CFP/YFP bacteria was estimated, with SCVs always being deleterious (see Fig. S3D), in accordance with: (i) SCVs having a growth disadvantage in RPMI (Fig. S5); (ii) all clones being streptomycin resistant (RpsL^K43R^).

### 2.7 Data analysis

All analyses were carried in R version 3.2.1, using *t*‐tests, Wilcoxon tests, ANOVA or linear mixed‐effects models (http://www.r-project.org/). Where required, data were transformed to meet assumptions made by parametric statistics.

### 2.8 Ethics statement

All experiments involving animals were approved by the Institutional Ethics Committee at the Instituto Gulbenkian de Ciência (project nr. A009/2010 with approval date 2010/10/15), following the Portuguese legislation (PORT 1005/92) which complies with the European Directive 86/609/EEC of the European Council.

## 3 Results and Discussion

### 3.1 Novel genetic targets, associated with the ETC, mediate the SCV phenotype in *E. coli*

Whole‐genome sequencing of 10 SCV clones evolved during interactions with MΦs and revealed a total of 19 mutations, with 1–3 mutations per clone (average: 1.9; Table 1). This is a large number of mutations, given that SCVs were isolated after only 60–120 generations of adaptation to MΦs. Interestingly, in half the populations (M1, M2 and M5), SCVs have distinct haplotypes and have therefore emerged independently. Thus, while we have not estimated the full genetic diversity within populations, it is clear that SCVs can be a polymorphic subpopulation, with different beneficial haplotypes in competition (a phenomenon known as clonal interference; Sniegowski & Gerrish, 2010).

The mutations in SCVs affected 11 different genetic targets, all in coding regions. To the best of our knowledge, 10 of these have not been previously associated with the SCV phenotype. The exception is *hemL,* the most common genetic target in our experiment: mutated in three of five independent populations. *hemL* has also been associated with SCVs in *S. enterica*, for spontaneous resistance to antimicrobial peptides (Pranting & Andersson, 2010) and during adaptation to the intracellular environment of fibroblasts (Cano et al., 2003). Additionally, we observed parallel mutations in two other genes (*yqiJ* and *yjbS*: mutated twice), and at the haplotype level with clones carrying mutations in *hemL*,* yqiJ* and *yjbS* being isolated from two different populations (M1 and M3). Importantly, functional annotation clustering (Table S2) and literature review on the function of the mutated genes revealed widespread functional parallelism, as all clones had at least one mutated gene associated with the ETC. Therefore, our WGS data show evidence for parallel adaptation at multiple levels.

Mutations in genes of the ETC are often associated with SCVs, however the diversity of mutational targets that can give rise to such SCVs is poorly understood. Our results (Table S2) indicate that the observed mutations target three classes of processes: first, oxidative phosphorylation, through mutations in subunit I of cytochrome bd oxidase (*cydA*; Green, Kranz, Lorence, & Gennis, 1984) and in both NADH:ubiquinone oxidoreductase I and II (*nuoG* and *ndh*, respectively, Young & Wallace, 1976; Weidner et al., 1993); second, heme and Fe‐S cluster synthesis through mutations in: (i) two enzymes that are involved in heme synthesis (*hemL* and *hemY*), which is a key prosthetic group in several enzymes of the ETC (e.g. cytochromes; Alefounder, Abell, & Battersby, 1988; Ilag, Jahn, Eggertsson, & Söll, 1991); (ii) *ygfZ*, which is thought to be involved in the synthesis/repair of Fe‐S clusters (present in enzymes as NADH:ubiquinone oxidoreductase I; Ote et al., 2006; Waller et al., 2010); and third, ubiquinone synthesis (the key electron acceptor for the ETC; Stroobant, Young, & Gibson, 1972), through mutations in an *O*‐methyltransferase (*ubiG*) and in *pepP*. While the function of *pepP* (an aminopeptidase) is not directly related to ETC (Yoshimoto, Murayama, Honda, Tone, & Tsuru, 1988), transposition of an insertion sequence (IS) into *pepP* is known to have polar effects on *ubiH* activity (part of the same operon; Way, Sallustio, Magliozzo, & Goldberg, 1999). We confirmed this result in our SCV clone, by demonstrating that (i) *ubiH* expression was strongly reduced in the *pepP* mutant (clone 41; Fig. S4A) and (ii) that this mutant was unable to grow in minimal media supplemented with succinate or glycerol, but not glucose (Fig. S4B and Appendix S1; this was previously known for *ubiH* mutants; Gupta, Mat‐Jan, Latifi, & Clark, 2000; Joyce et al., 2006). Importantly, revertant clones, derived from clone 41 (i.e. producing a large colony morphotype), were able to grow on succinate and glycerol (Fig. S4C). As *ubiH* is involved in ubiquinone synthesis (Young, Stroobant, Macdonald, & Gibson, 1973), these data indicate that the SCV phenotype of the *pepP* mutant is also mediated by reduced activity of the ETC (rather than a direct effect of disrupting *pepP* function).

The two positive control clones, which do not show an SCV phenotype, (i.e. form large colonies; Fig. S1), carry a single mutation in *mscM,* which encodes a mechanosensitive ion channel of miniconductance. This is potentially important under hypo‐osmotic shock (Edwards et al., 2012), but is not associated with the ETC. Therefore, these results generally suggest that the SCV phenotype that emerged during *E. coli* adaptation to MΦs is mediated by defects in the ETC, as has often been observed for SCVs isolated from experiments or clinical samples, across multiple species (Cano et al., 2003; Dean et al., 2014; Pranting & Andersson, 2010; Proctor et al., 2006; Roggenkamp et al., 1998).

### 3.2 *E. coli* SCVs have phenotypes similar to SCVs from clinical isolates

We measured several phenotypes that are typically observed in SCVs with mutations in the ETC (both in clinical and experimental isolates; results summarized in Table S3). We found that SCVs had a generally high (but variable) reversion frequency (to large colony phenotype), ranging from 10^−2^ to ~10^−6^. In eight of 10 clones, the reversion frequency was >10^−5^, in line with previous observations (Häussler et al., 1999; Proctor et al., 2006; Roggenkamp et al., 1998; Trülzsch et al., 2003). When tested for catalase activity, four SCV clones were catalase negative (Proctor et al., 2006), all of which carried mutations in *hemL* (clones 11, 26, 28, 61; Table S3). This is in agreement with the fact that heme is a co‐factor in both *E. coli* catalases (Schellhorn, 1995).

Small‐colony variants typically show poor growth under in vitro conditions, as a result of auxotrophy for specific compounds (Proctor et al., 2006; Wei et al., 2011). In agreement, our SCVs generally reached a lower carrying capacity than the ancestral strain, both in poor (M9glu0.4) and rich media (RPMI; Table S3; Fig. S5). However, the effect of the SCV phenotype on growth rate was media‐dependent. In poor media, the relative growth rate was generally lower (8/10 SCV clones), but in rich media, this was strongly dependent on the SCV clone. Thus, it is important to understand how the within‐host environment affects SCV growth in order to be able to predict the in vivo fitness of this morphotype (this could be done through indirect methods as in Barroso‐Batista et al. 2015).

Two of the auxotrophies that are often observed in SCVs with mutations in the ETC are for hemin and aminolevulinic acid (5‐ALA; Proctor et al., 2006; Pranting & Andersson, 2010). Accordingly, supplementation of our SCV clones with either of these compounds improved their growth (Table S3; Fig. S6). However, while the benefit provided by hemin was observed across all clones and growth parameters (lag phase, maximum growth rate and carrying capacity), 5‐ALA specifically shortened the lag phase of only five clones, four of which carry mutations in *hemL* (~50% reduction). This is consistent with the described role of *hemL* in converting glutamate‐1‐semialdehyde to 5‐ALA (Ilag et al., 1991), the first committed precursor of heme‐biosynthesis, a key co‐factor in several proteins of the ETC (Ilag et al., 1991).

These results highlight the pleiotropic consequences of the SCV phenotype. Moreover, while SCVs show qualitative convergence for slow growth and high reversion frequency, the phenotypes are quantitatively variable across SCV clones. The only clones that can be qualitatively distinguished are the *hemL* mutants due to their 5‐ALA auxothrophy and negative catalase test.

### 3.3 SCVs have a fitness advantage in the intracellular environment of macrophages

**Figure 1:**
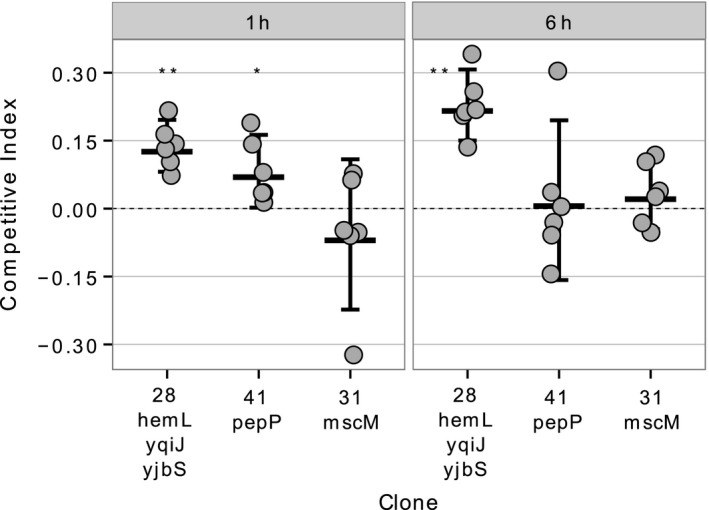
SCVs have a fitness advantage inside MΦs. Competitive index (CI) for two SCV clones (28, 41) and a positive control clone (31), inside MΦs at 1 hr (left) and 6 hr (right) postinfection (YFP ancestral is used as reference). Dots represent the CI for each independent experiment (*n* = 6), and lines represent mean ± 2 standard errors (*SE*), across all experiments. Significant deviations from CI = 0 are denoted by *(*p < *.05) or **(*p < *.01, t‐test). See Figure S2 for evidence of marker neutrality

As the SCVs used in this study emerged in the presence of MΦs, we carried out competition assays to test whether SCVs have a fitness advantage inside MΦs. Figure 1 shows that an SCV clone carrying mutations in *hemL, yqiJ* and *yjbS* had a fitness advantage (CI significantly different from 0) after both short (1 hr) and prolonged (6 hr) residence inside MΦs (at 1 hr: CI = 0.14 ± 0.06, mean ± 2 standard errors, *t*_5_ = 6.84, *p *<* *.01; at 6 hr: CI = 0.22 ± 0.08, *t*_*5*_ = 8.25, *p *<* *.001). A clone with an IS1 insertion in *pepP* displayed a transient fitness advantage, visible at 1 hr (CI = 0.08 ± 0.08, *t*_5_ = 2.9, *p* = .03), but not at 6 hr. As the ancestral strain of *E. coli* does not replicate inside MΦs (as most commensal strains; Koli, Sudan, Fitzgerald, Adhya, & Kar, 2011), the observed fitness advantage of these SCVs is likely mediated by an increased survival ability of SCVs inside MΦs. We also tested a clone with an *mscM* mutation that shows a colony size similar to the ancestral and observed no change in competitive ability. Overall, these data show that SCVs have increased intracellular survival in MΦs, providing an explanation for their repeated emergence during experimental adaptation of *E. coli* to MΦs (Miskinyte et al., 2013). This is also in agreement with studies showing that SCVs can emerge and have a fitness advantage in the intracellular milieu of nonprofessional phagocytes (Cano et al., 2003; Tuchscherr et al., 2011; Vesga et al., 1996).

### 3.4 SCVs present antagonistic pleiotropy for antibiotic resistance in vitro

**Figure 2:**
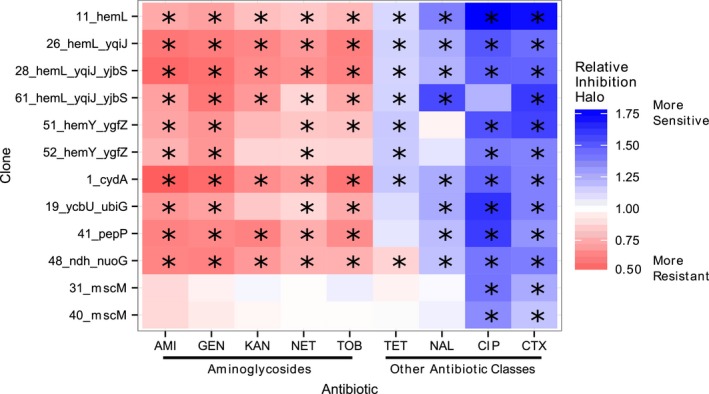
SCVs show increased resistance to aminoglycosides, but collateral sensitivity to other antibiotic classes. Heatmap of the relative inhibition halo (i.e. mean inhibition halo for SCV clones relative to that of the ancestral). In total, 3–8 independent replicates were carried for any clone‐antibiotic combination. Colours red, blue and white indicate whether the inhibition halo is smaller (i.e. more resistant), larger (i.e. more sensitive) or equal to the ancestral, respectively. Significant differences (*p *< .05; anova contrasts) relative to the ancestral are denoted by a “*”. AMI – Amikacin; GEN – Gentamicin; KAN – Kanamycin; NET – Netilmicin; TOB – Tobramycin; TET – Tetracycline; NAL – Nalidixic acid; CIP – Ciprofloxacin; CTX – Cefotaxime. See Fig. S7 for the mean (±2*SE*) relative inhibition halo and the mean halo (in mm)

A common phenotype of SCVs is increased resistance to aminoglycoside antibiotics (von Eiff et al., 1997; Musher et al., 1977). However, much less is known about the antibiotic resistance profile of SCVs against nonaminoglycoside antibiotics (Cano et al., 2003; Pranting & Andersson, 2010). In Fig. 2, we show that *E. coli* SCVs were generally more resistant to aminoglycosides, but were more susceptible to tetracycline, nalidixic acid and ciprofloxacin (quinolones), and cefotaxime (a β‐lactam). All SCV clones had significantly increased resistance to at least three aminoglycosides (relative to the ancestral), but also increased susceptibility to at least three nonaminoglycosides (*p < *.05 ANOVA contrasts; see Fig. S7 for mean ± 2*SE*). The positive control clones (*mscM* mutants), whose colony size is similar to the ancestral, do not show such a pattern.

Recent work, using in vitro experimental evolution of *E. coli* in the presence of aminoglycosides, has shown that resistant mutants often have increased sensitivity to other antibiotics (including nalidixic acid, ciprofloxacin or tetracycline; Imamovic & Sommer, 2013; Lázár et al., 2013; Oz et al., 2014; Suzuki, Horinouchi, & Furusawa, 2014), a phenomenon termed collateral sensitivity (Szybalski & Bryson, 1952). Interestingly, some of these studies recurrently observed mutations in genes with similar functions to those observed here (e.g. heme synthesis: *hemA*; NADH dehydrogenase I: *nuoF* and cytochrome bd‐I terminal oxidase: *cydA*; Lázár et al., 2013; Oz et al., 2014; Suzuki et al., 2014), suggesting that adaptation to aminoglycosides may also lead to bacteria that are more adapted to macrophages (Durão, Güleresi, Proença, & Gordo, in press; Miskinyte & Gordo, 2013).

### 3.5 SCVs have a fitness advantage in vivo that can be reversed with tetracycline treatment

While several recent papers have shown collateral sensitivity in vitro (Imamovic & Sommer, 2013; Lázár et al., 2013; Roemhild, Barbosa, Beardmore, Jansen, & Schulenburg, 2015)*,* whether this phenomenon also occurs in vivo is currently unknown. As the effect of resistance mutations is highly dependent on the environment (Miskinyte & Gordo, 2013; Trindade, Sousa, & Gordo, 2012), it is imperative to determine whether collateral sensitivity is also observed in vivo. Here, we used the streptomycin‐treated mouse model of gut colonization to test for the competitive ability of SCVs against the ancestral, in the presence or absence of treatment with kanamycin, nalidixic acid or tetracycline.

**Figure 3:**
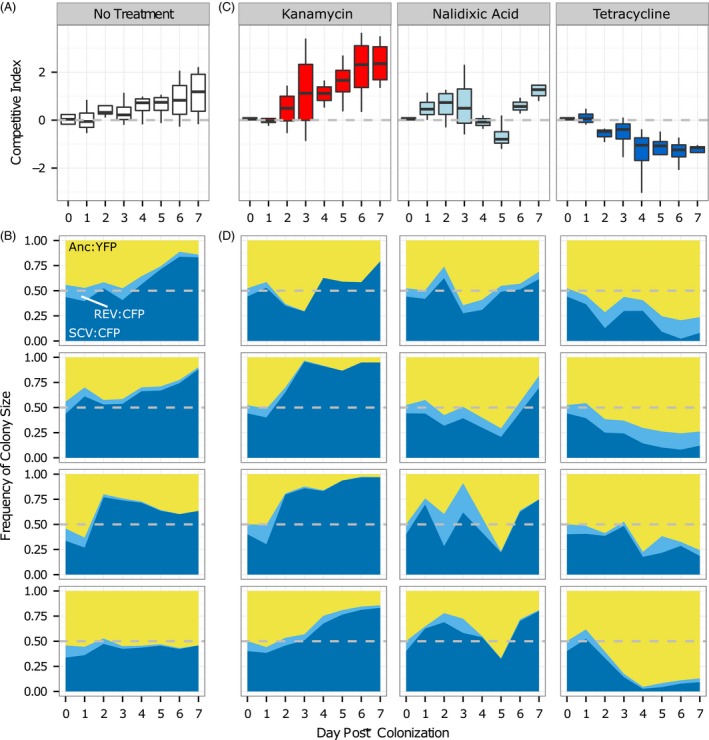
The fitness advantage of SCVs within the mouse gut is modulated by antibiotic treatment. (A and C) Boxplots of the competitive index (CI) of SCVs (CFP) against the ancestral (YFP), during colonization of the mouse gut in mice that did not received antibiotic treatment (A) or in mice that were treated (C) with kanamycin (left), nalidixic acid (centre) or tetracycline (right). CI was calculated as the natural logarithm of the ratio of CFP cfu to YFP cfu. (B and D) Frequency of fluorescent marker/colony size for mice without (B) or with (D) antibiotic treatment. Each panel represents a single mouse and shows the frequency of SCVs (SCV:CFP; dark blue), SCV revertants (REV:CFP; light blue) and ancestral bacteria (Anc:YFP; yellow). Horizontal dashed lines in A–D indicate where SCVs and Anc are competitively equivalent. See Fig. S3 for slopes of CI against time, mean (±2*SE*) CFP frequency and bacterial loads

Remarkably, we observed that SCVs had a fitness advantage in the mouse gut (Fig. 3A,B): SCV frequency increased, and by day 7, SCVs constituted the majority of the population in three of four mice. Accordingly, SCV ratio significantly increased with time (Fig. 3A; $\chi_{1}^{2}$ *= *11.4, *p < *.001), with a positive selection coefficient (per day) of 0.14 (±0.05, standard error; *SE*; see methods; (Dykhuizen, 1990); see Fig. S3 for slopes). Conversely, the frequency of revertant bacteria (forming large CFP colonies) decreased from ~0.12 on day 0 to below 0.03 by day 7 post‐colonization (Fig. 3B). These results show that SCVs can have an increased competitive ability in a natural environment for *E. coli*. Interestingly, *E. coli* SCVs have been isolated from the gut in natural infections of humans (Trülzsch et al., 2003) and during adaptation to the mouse gut (Lee et al., 2010).

In agreement with the in vitro results, SCV fitness in the mouse gut was dependent on the antibiotic treatment (time:treatment interaction: $\chi_{3}^{2}$ *= *58.9, *p < *.0001). SCV fitness significantly increased with kanamycin treatment ($\chi_{1}^{2}$ *= *10.2, *p < *.01), leading to a selection coefficient of 0.36 (±0.05, *SE*). On the other hand, tetracycline treatment, significantly reduced SCV fitness within the mouse gut ($\chi_{1}^{2}$ *= *28.8, *p < *.0001), leading to a fitness disadvantage (selection coefficient: −0.23 ± 0.05, *SE*). Nalidixic acid also led to a reduction in the fitness advantage of SCVs (slope: 0.05 ± 0.05, *SE*). However, this difference was not significant ($\chi_{1}^{2}$ *= *1.9, *p* = .17).

As far as we are aware, these results provide the first in vivo demonstration that the frequency of SCVs can be increased by aminoglycoside treatment, but decreased by tetracycline due to their collateral sensitivity to this antibiotic. Furthermore, while we observed that antibiotics strongly influence the composition of the *E. coli* population, the total population size remains relatively stable (Fig. S3), albeit lower than in mice drinking only streptomycin‐treated water ($\chi_{1}^{2}$ *= *4.8, *p* = .03). As commensal bacteria can acquire pathogenic traits through just a few mutations (Koli et al., 2011; Limoli et al., 2014; Miskinyte et al., 2013), these results suggest that it may be possible to reduce the frequency of bacteria with such traits (e.g. SCVs) without eliminating the commensals.

## 4 Conclusions

Small‐colony variants are an important bacterial morphotype often associated with chronic infections and antibiotic resistance. In this manuscript, we identified new mutational paths that confer an SCV phenotype and show that these mutations have multiple pleiotropic phenotypic consequences in vitro and in vivo (in the mouse gut). Thus, our results highlight that combining experimental evolution in the presence of key cells of the innate immune system, with WGS and phenotypic tests can reveal: (i) the genetic basis of bacterial traits important for within‐host fitness and (ii) the selective pressures that lead to such traits.

Importantly, the SCV phenotype leads to a trade‐off, both in vitro and in vivo, between increased aminoglycoside resistance and collateral sensitivity to other antibiotic classes. As SCVs carry multiple mutations in genes related to the ETC, collateral sensitivity is likely mediated by a reduction in proton‐motive force (PMF) across the inner membrane. This is because resistance to other antibiotic classes is mediated by several PMF‐dependent efflux pumps, thus reduced PMF can lead to increased sensitivity to some antibiotics (Lázár et al., 2013).

Collateral sensitivity has been suggested as an important principle for the design of treatment regimens that curb the evolution of antibiotic resistance (Imamovic & Sommer, 2013; Lázár et al., 2013). However, at the moment, we are aware of only a single report using this concept to treat bacteria in vivo (Gonzales et al., 2015). Thus, more work is currently needed to establish the in vivo effectiveness of therapies involving collateral sensitivity (Baym, Stone, & Kishony, 2016).

## Data Archiving Statement

Data available from the Dryad Digital Repository: http://dx.doi.org/10.5061/dryad.3h9f5
