# Supplementary material for: Macrophage adaptation leads to parallel evolution of genetically diverse Escherichia coli small‐colony variants with increased fitness in vivo and antibiotic collateral sensitivity
Source: Evol Appl. 2016 Jun 30;9(8):994–1004. doi: 10.1111/eva.12397 (PMC4999529; doi:10.1111/eva.12397)
Supplement: Supplementary file 1 [file EVA-9-0994-s001.docx]

**Appendix**

Supporting Information to:

Macrophage adaptation leads to parallel evolution of genetically diverse *Escherichia coli* small-colony variants with increased fitness *in-vivo* and antibiotic collateral sensitivity

Document contents:

- **Supplementary Methods**
  - *Summary of experiment on bacterial adaptation to MΦs*
  - *qPCR and phenotypic tests to confirm the polar effect of* pepP::IS1 *(clone 41) on the expression and function of* ubiH
  - *Assays for measuring the phenotypic traits of SCVs*
  - *MΦ culture conditions, seeding and activation*
- **Supplementary Figure Legends**

**Supplementary Methods**

*Summary of experiment on bacterial adaptation to MΦs*

This experiment is described in (Miskinyte *et al.*, 2013). Briefly, 12 populations were founded from a single clone of MC4100-CFP. This clone expresses cyan fluorescent protein (CFP) integrated at the *galK* locus, carries an ampicillin resistance cassette and a mutation in *rpsL* (K43R), which makes it resistant to streptomycin. The 12 populations evolved in RPMI 1640 supplemented with heat-inactivated FCS (10%), L-glutamine (2 mM), sodium pyruvate (1 mM), hepes (10 mM), 2-mercaptoethanol solution and streptomycin (100μg/ml). Hereafter this media is referred to as RPMI-Strep. Of the 12 populations, 6 evolved in the presence of MΦs (MΦ lines) and another 6 in its absence (control lines). For the MΦ lines, 10^6^ bacteria were incubated with 10^6^ MΦs (murine cell-line RAW 264.7) per well, in a 12-well plate, with 3ml RPMI-Strep. MΦs were activated for 24h before infection with 2mg/ml CpG-ODN 1826 (in RPMI-strep). 24h post-infection, MΦs were detached and the whole-culture was centrifuged (4000rpm, 10min.) to pellet cells and lyse MΦs, thereby releasing intracellular bacteria. This pool of intra- and extracellular bacteria was then FACS-counted and 10^6^ bacteria were incubated with newly activated MΦs. This procedure was repeated daily for 30 days. A similar procedure was used for the control lines, except for the absence of MΦs and for the fact that 10^4^ bacteria were passaged (this ensured that the same number of bacterial generations had elapsed, per day, in both control and MΦ lines). Using this procedure, the emergence of small-colony variants and mucoid colonies was observed in populations evolving in the presence of MΦs. In the control bacterial populations, evolving without MΦs, diversity for colony morphology was not observed.

*RT-qPCR and phenotypic tests to confirm the polar effect of* pepP::IS1 *(clone 41) on the expression and function of* ubiH

RT-qPCR: Ancestral and SCV clone 41 were streaked in LB-agar plates and incubated overnight. Five colonies from the ancestral or 10 SCV colonies were separately pooled and inoculated into 2 ml RPMI and incubated at 37ºC, with 5% CO_2_, until OD_600nm_ reached 0.5. This procedure was repeated five times, resulting in five independent replicates. The whole culture was then centrifuged at the maximum speed, for 5 min. (at 4ºC) and the pellet was ressuspended in lysozyme solution (5 mg lysozyme /ml DEPC treated water, Sigma protocol) and incubated at 37ºC for 30 minutes. This promotes disruption of the bacterial cell wall, allowing for RNA extraction.

RNA was extracted with the Qiagen RNeasy Mini Kit (RNA concentration and quality evaluated with Nanodrop 2000). DNase treatment was done with the RQ1 DNase (Promega), according to the manufacturer’s protocol. The resulting RNA was used for the reverse transcription, by mixing with 1 µg of RNA, with 0.5 µl random primers and DEPC-water (final volume of 17 µl), followed by incubation at 70ºC for 5min. Afterwards the M-MLV Reverse Transcriptase Protocol (Promega) was carried, by adding the following to the first mix: RT buffer (5 µl), RT enzyme (0.5 µl) and dNTP mix (2 µl). This was then incubated for 10 min at 25ºC, 50min at 50ºC and 10 min at 70ºC.

We used a relative quantification method of analysis with normalization against a reference gene. qPCR was executed in BioRad CFX 384 with itaq universal sybr green supermix (BioRad). cDNA was diluted 10-fold before use in the RT-qPCR. The qPCR reaction conditions were: 39 cycles of 10 min at 95ºC, 30 sec at 95ºC, 1 min at 60ºC and finally 30 s at 72ºC. Melting curve analysis was performed to verify product homogeneity. All reactions included three technical replicates for each sample. Data were normalized by the Pfaffl method (Pfaffl, 2001) using the *ssrA* housekeeping gene of *E. coli* as a reference (Zhou *et al.*, 2011). Primers for *ubiH* were: 5’-CAGCAGGAGCCTTACGAACA-3’ (forward) and 5’-CTCGTCACTCCACGACAACA-3’ (reverse). Primers for *ssrA* were: 5’-ACGGGGATCAAGAGAGGTCAAAC-3’ (forward) and 5’-CGGACGGACACGCCACTAAC-3’ (Zhou *et al.*, 2011).

Phenotypic tests: Bacteria were plated in LB-agar plates and incubated overnight (37ºC). SCV or Ancestral colonies were then picked, resuspended in 200µl PBS and five independent streaks (per clone, per media) were then made in minimal media with 0.4% glucose, glycerol or succinate and incubated at 37ºC for 48h. The same results were obtained for all replicates and a representative example is shown in Figure S4B. For the plates with SCV clone 41 and its large colony revertant (Figure S4C), three independent SCV colonies were picked and grown overnight in RPMI (37ºC, 5% CO_2_). These cultures were then plated and pairs of revertant and SCV from the same plate were resuspended, streaked and incubated as described above.

*In-vitro growth assays*

Reversion frequency: The reversion frequency from SCV to large-colony phenotype (revertant) was determined as described in (Roggenkamp *et al.*, 1998; Miskinyte *et al.*, 2013) (4 independent replicates per clone).

Catalase activity test: bacterial colonies were added to glass slides, on top of which 3% H_2_O_2_ was added (Roggenkamp *et al.*, 1998). Clones were scored positive if intense bubble formation occurred within the first 5s (15 independent colonies per clone). Bubble formation was not observed for the Δ*rpoS* strain (negative control; (Al Mamun *et al.*, 2012).

Growth parameters in rich (RPMI) and poor media (M9 minimal media with 0.4% glucose): Growth curve parameters for the ancestral strain (MC4100-CFP) and each of the sequenced clones were estimated by measuring OD_600nm_ (at 37ºC) with a Bioscreen C system (Oy Growth Curves Ab) over 48h. Growth assays were carried according to the conditions described in the Appendix. For each clone, a total of 4 independent colonies were tested (up to 7 replicates/colony). The R package *grofit* (Kahm *et al.*, 2010), with the spline method, was used to estimate maximum growth rate and carrying capacity. As we have multiple OD measurements in stationary phase, carrying capacity is calculated as the maximum value of the fitted spline. Relative growth parameters were calculated by dividing parameter estimates for each clone, by the estimate for the ancestral.

Hemin and Aminolevulinic-acid (5-ALA) auxothrophy: Growth assays were performed (as described above) in M9glu0.4 supplemented or not with either hemin (50μg/ml) or 5-ALA (50μg/ml) and *grofit* (Kahm *et al.*, 2010) was used to estimate the lag phase, maximum growth rate and carrying capacity. Growth parameters with hemin or 5-ALA were standardized relative to those obtained in the absence of these compounds, for each independent colony (4 independent colonies, up to 3 replicates per colony).

Bioscreen set up: Growth measurements were taken in Bioscreen, every 15min, with 5s shaking before each measurement. For RPMI, wells were covered with oil to avoid the formation of air bubbles, which interfere with the OD_600nm_ reading. To ensure that SCVs were added to the wells (and not revertant colonies), SCV colonies were picked from agar plates, resuspended in 300μl PBS. 10μl of this suspension was then added to each well.

*MΦ culture conditions, seeding and activation*

The RAW 264.7 murine MΦ cell line was maintained in an atmosphere containing 5% CO_2_ at 37°C in RPMI 1640 (Gibco) supplemented with 2mM L-glutamine (Invitrogen), 1mM sodium pyruvate (Invitrogen), 10mM hepes (Invitrogen), 100μg/ml streptomycin (Gibco), 50µM 2-mercaptoethanol solution (Gibco), with 10% heat-inactivated FCS (Gibco).

For the competition experiments, MΦs (~10^5^) were seeded in 1ml RPMI (in 24-well plates) and incubated for 20h, at 37ºC with 5% CO_2_. The media was then replaced with RPMI containing 2µg/ml CpG-ODN 1826 (5′TCCATGACGTTCCTGACGTT 3′ - Sigma), to activate MΦs and these were incubated at 37ºC with 5% CO_2_ for 24h.

**Supplementary Figure Legends**

**Figure S1. Representative colonies of all the evolved clones and the Ancestral.** Bacteria were plated on LB agar and incubated at 37ºC, for 24h. The picture of an isolated representative colony is shown and clone numbers are given above each colony.

**Figure S2. Fluorescent markers are neutral inside MΦs.**

Competitive index (CI) for Ancestral CFP/YFP, inside MΦs at 1h (left) and 6h (right) post-infection. CI is not significantly different from zero at 1h (t_4_ = 0.43, p = 0.69) or 6h (t_4_ = 1.47, p = 0.22), indicating that the fluorescent markers are neutral. Dots represent the CI for each independent experiment (n=5) and lines represent mean ± 2 standard errors (SE), across all experiments.

**Figure S3. Fluorescent marker and bacterial load dynamics during colonization of the mouse gut.**

(A) Four mice were colonized with a mixture of isogenic CFP and YFP MC4100 (Ancestral strain), to test for marker neutrality. In the majority of the mice, marker frequency remained very close to 0.5 (each panel represents one mouse; blue – Ancestral-CFP; yellow – Ancestral-YFP).

(B) The competitive index for the Ancestral-CFP vs. Ancestral-YFP is not significantly affected by time, across the 7 days (*χ^2^_1_=*0.82, *p=*0.36), indicating that the fluorescent markers are neutral. Competitive index was calculated as natural logarithm of the ratio of CFP cfu to YFP cfu, against time.

(C) Linear regressions of the competitive index along time that were used to calculate the selection coefficients (i.e. slopes) presented in the results (line colours are the same as for those in Figures 3A and 3C).

(D) *In vitro* competitive index of SCVs (against the ancestral), at different streptomycin concentrations. Competitions were done by inoculating a 50:50 mixture of the SCV population used *in-vivo* and the YFP ancestral into RPMI at 100μg/ml or 500μg/ml of streptomycin (n=3 replicates per concentration). 24h post-incubation the frequency of CFP/YFP bacteria was estimated and the competitive index calculated. SCVs are deleterious at both streptomycin concentrations, but that the fitness cost is higher at 100μg/ml. This indicates that the observed advantage for SCVs is not due to streptomycin treatment, as would be suspected since all clones carry the streptomycin resistance mutation RpsL^K43R^. Consistent with this conclusion, we note that: i) Hentges *et al.* (1985) estimated the concentration of streptomycin at 180 μg/g in cecal contents for mice treated with the same streptomycin concentration that we used; ii) if the advantage of SCVs in the mouse gut was due to streptomycin, we would expect SCV frequency to increase immediately after gavage, but we only see this increase after the first 2-3 days of gut colonization.

(E-F) Mean marker frequency and Log_10_ bacterial loads (±2SE) observed during colonization of the mouse gut (differently coloured lines represent different mice). These allow an assessment of the variation in these measurements, for the data used for Figures 3 and S3A-B. (E and F-top rows) CFP frequency. (E and F-bottom row) Log_10_ bacterial loads per g of feces. When these are at 0, no bacteria could be detected on the stock fecal suspension. (E-left) Mice colonized with a mixture of SCV-CFP and Ancestral-YFP. (E-right) Mice colonized with a mixture of Ancestral-CFP and Ancestral-YFP. Mice colonized with a mixture of SCV-CFP and Ancestral-YFP and treated with: (F-left) kanamycin; (F-centre) nalidixic acid; (F-right) tetracycline.

**Figure S4. RT-qPCR and phenotypic tests confirm the polar effect of *pepP::*IS1 on the expression and function of *ubiH*.**

(A) Log_2_ fold change of *ubiH* gene expression, measured by RT-qPCR, is significantly reduced in the *pepP* mutant (clone 41; t_4_=-24.97, *p*<0.001) relative to the Ancestral clone (Pfaffl, 2001; Zhou *et al.*, 2011), across 5 independent biological replicates.

(B-C) Streaks in M9 minimal media with 0.4% glucose, glycerol or succinate of: (B) *Escherichia coli* MC4100-CFP Ancestral (Anc) and SCV clones 41 (*pepP::*IS1) and 19 (*ubiG::*Δ10bp and *ycbU::*IS2); (C) SCV clone 41 (SCV) and its revertant (Rev), which forms large colonies. Mutants in *ubiH* or *ubiG* are known to be unable to grow in minimal media supplemented with glycerol or succinate (Hsu *et al.*, 1996; Gupta *et al.*, 2000; Joyce *et al.*, 2006). Black arrows point to SCV growth. Clone 19 is used here as a positive control as this clone has a 10bp deletion in *ubiG*, involved in the same pathway as *ubiH*, which leads to ubiquinone production. Five (B) or three (C) independent replicates were carried, all of which revealed the same result.

**Figure S5. SCVs have a growth disadvantage in rich (A, C) and poor media (B,D).** In minimal media (M9) this disadvantage is observed for most clones, both for the growth rate and for the carrying capacity. On the other hand, in RPMI, this disadvantage is most obvious for the carrying capacity. Boxplots represent the growth rate (or carrying capacity) relative to the mean values obtained for the Ancestral. * indicates a significant difference (*p<0.05*; anova contrasts), relative to the ancestral.

**Figure S6. Effects of 5-ALA (A-C) or hemin (D-F) on the growth of the Ancestral and the sequenced clones.** For each clone, the relative lag phase (A,D), growth rate (B,E) and carrying capacity (C,F) in the presence of 5-ALA (or hemin) was standardized relative to the mean values obtained in the absence of these compounds. Therefore, any significant deviations from 1, indicate that these compounds affect growth and are highlighted by a * (*p<0.05*, anova contrasts). While the effect of 5-ALA is growth phase and clone dependent, hemin generally improved growth, even of the ancestral clone. Therefore, insets for the mean relative growth parameters, in the presence of hemin, are shown for the ancestral and as a mean of all SCVs. These indicate that the effect of hemin on the growth rate and carrying capacity is substantially larger for SCVs than for the Ancestral.

**Figure S7. Mean relative inhibition halo (±2SE) for all tested antibiotics.** Numbers inside each bar indicate the average inhibition halo, measured in mm.

**References**

Al Mamun, A.A.M., Lombardo, M.-J., Shee, C., Lisewski, A.M., Gonzalez, C., Lin, D., *et al.* 2012. Identity and Function of a Large Gene Network Underlying Mutagenic Repair of DNA Breaks. *Science* **338**: 1344–1348.

Gupta, S., Mat-Jan, F., Latifi, M. & Clark, D.P. 2000. Acetaldehyde dehydrogenase activity of the AdhE protein of Escherichia coli is inhibited by intermediates in ubiquinone synthesis. *FEMS Microbiol. Lett.* **182**: 51–55.

Hentges, D.J., Stein, A.J., Casey, S.W. & Que, J.U. 1985. Protective role of intestinal flora against infection with Pseudomonas aeruginosa in mice: influence of antibiotics on colonization resistance. *Infect. Immun.* **47**: 118–122.

Hsu, A.Y., Poon, W.W., Shepherd, J.A., Myles, D.C. & Clarke, C.F. 1996. Complementation of coq3 mutant yeast by mitochondrial targeting of the Escherichia coli UbiG polypeptide: evidence that UbiG catalyzes both O-methylation steps in ubiquinone biosynthesis. *Biochemistry (Mosc.)* **35**: 9797–9806.

Joyce, A.R., Reed, J.L., White, A., Edwards, R., Osterman, A., Baba, T., *et al.* 2006. Experimental and computational assessment of conditionally essential genes in Escherichia coli. *J. Bacteriol.* **188**: 8259–8271.

Kahm, M., Hasenbrink, G., Lichtenberg-Fraté, H., Ludwig, J. & Kschischo, M. 2010. grofit: fitting biological growth curves with R. *J. Stat. Softw.* **33**: 1–21.

Miskinyte, M., Sousa, A., Ramiro, R.S., de Sousa, J.A.M., Kotlinowski, J., Caramalho, I., *et al.* 2013. The Genetic Basis of Escherichia coli Pathoadaptation to Macrophages. *Plos Pathog.* **9**: e1003802.

Pfaffl, M.W. 2001. A new mathematical model for relative quantification in real-time RT-PCR. *Nucleic Acids Res.* **29**: e45.

Roggenkamp, A., Sing, A., Hornef, M., Brunner, U., Autenrieth, I.B. & Heesemann, J. 1998. Chronic prosthetic hip infection caused by a small-colony variant of Escherichia coli. *J. Clin. Microbiol.* **36**: 2530–2534.

Zhou, K., Zhou, L., Lim, Q. ’En, Zou, R., Stephanopoulos, G. & Too, H.-P. 2011. Novel reference genes for quantifying transcriptional responses of Escherichia coli to protein overexpression by quantitative PCR. *BMC Mol. Biol.* **12**: 18.
