# Supplementary figures and images for: Macrophage adaptation leads to parallel evolution of genetically diverse Escherichia coli small‐colony variants with increased fitness in vivo and antibiotic collateral sensitivity
Source: Evol Appl. 2016 Jun 30;9(8):994–1004. doi: 10.1111/eva.12397 (PMC4999529; doi:10.1111/eva.12397)

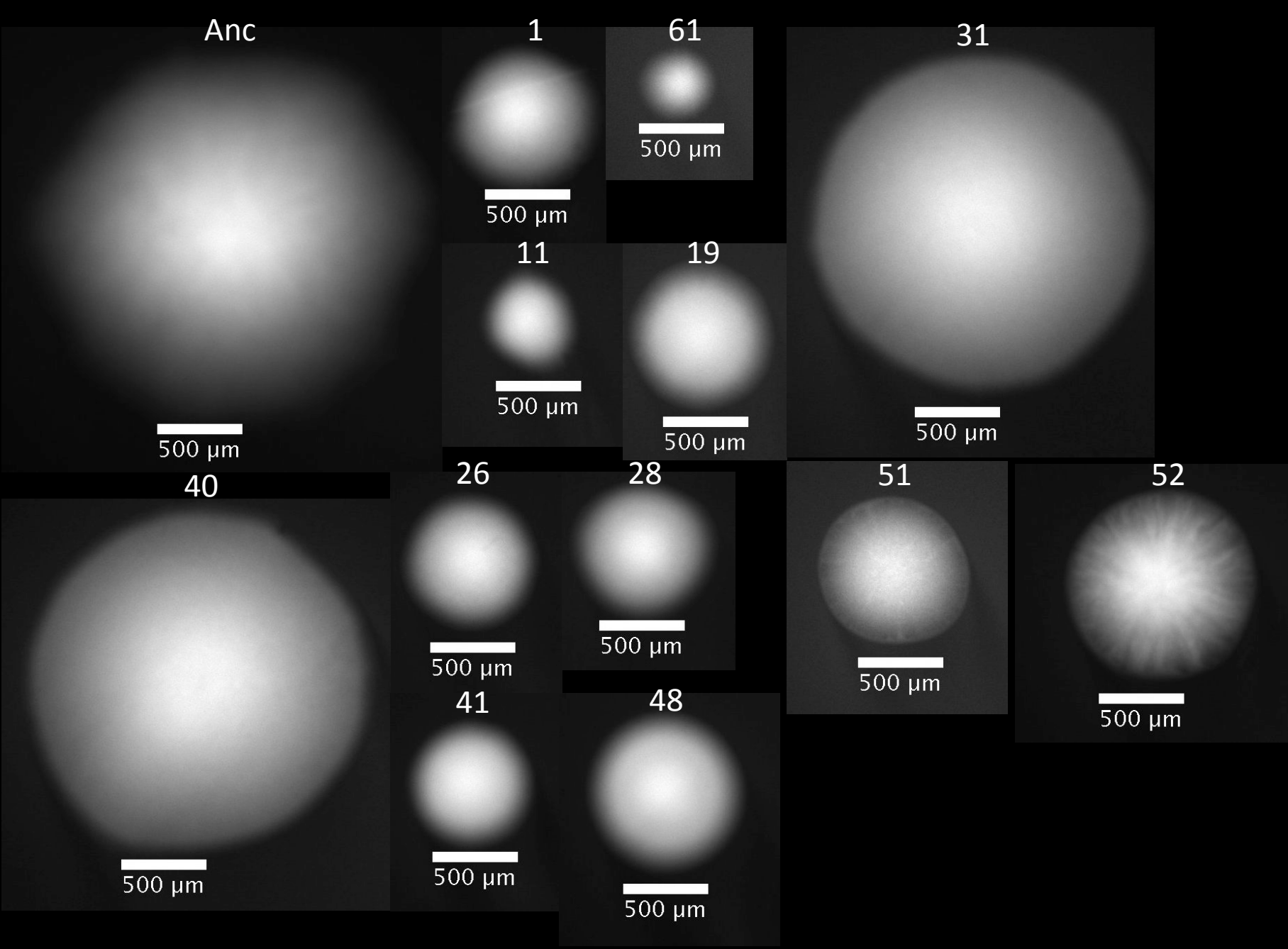

Supplement: Supplementary file 2 [file EVA-9-0994-s002.pdf]

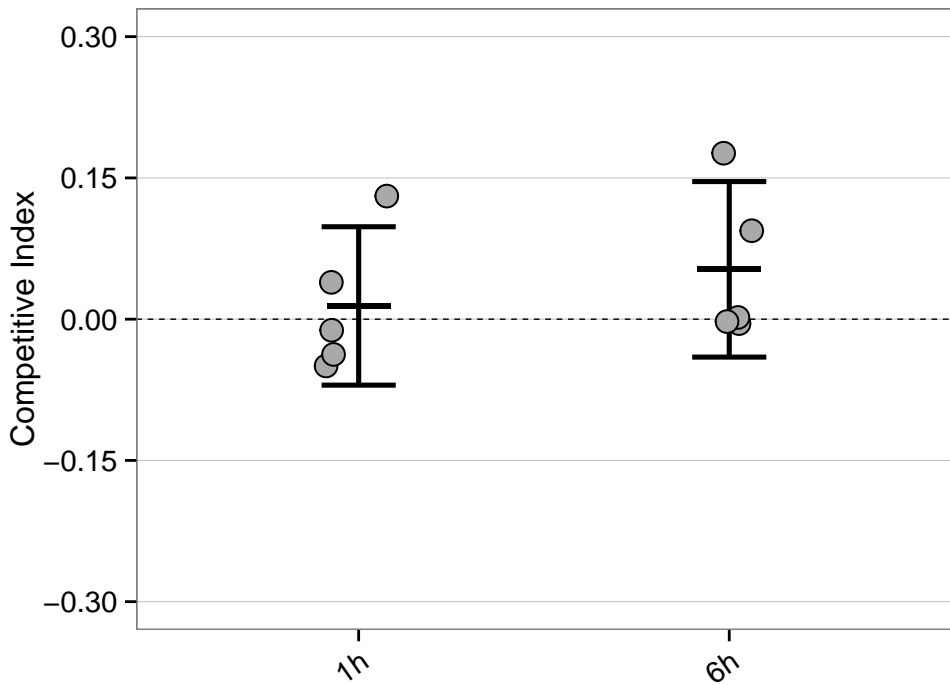

Supplement: Supplementary file 3 [file EVA-9-0994-s003.pdf]

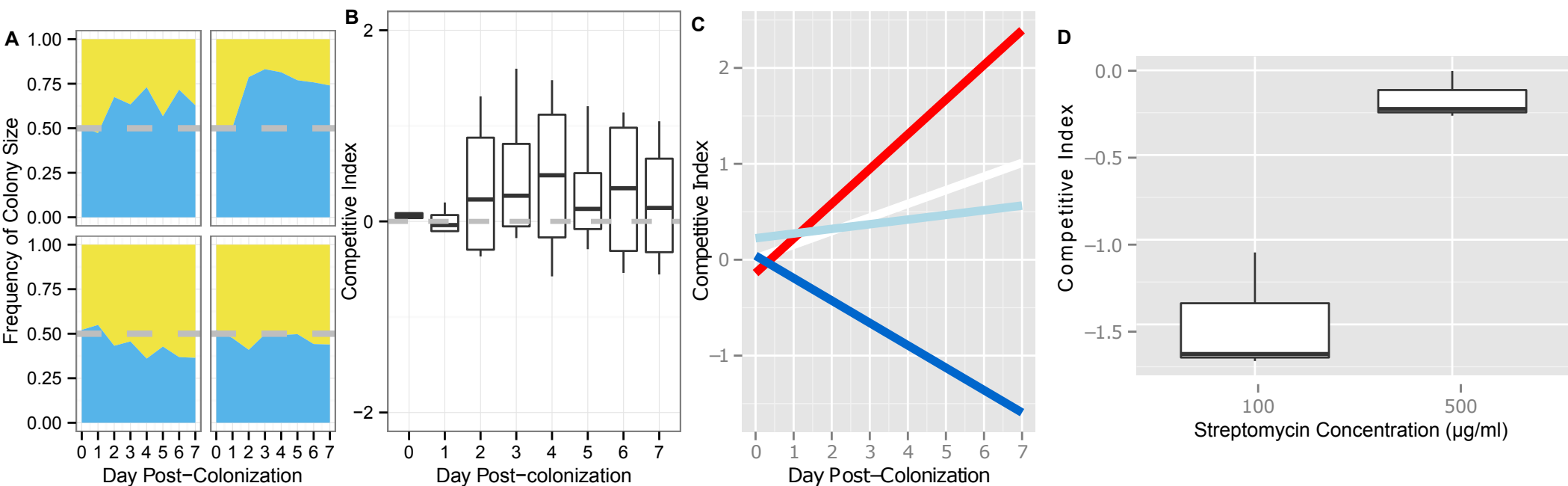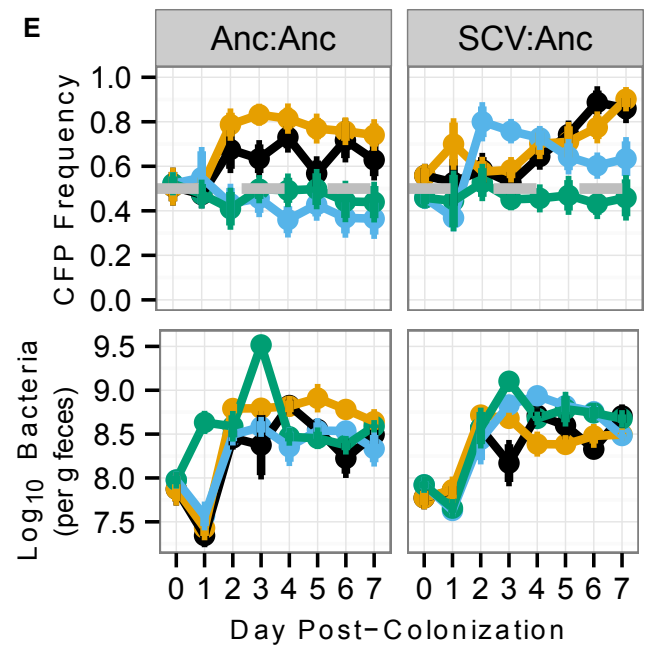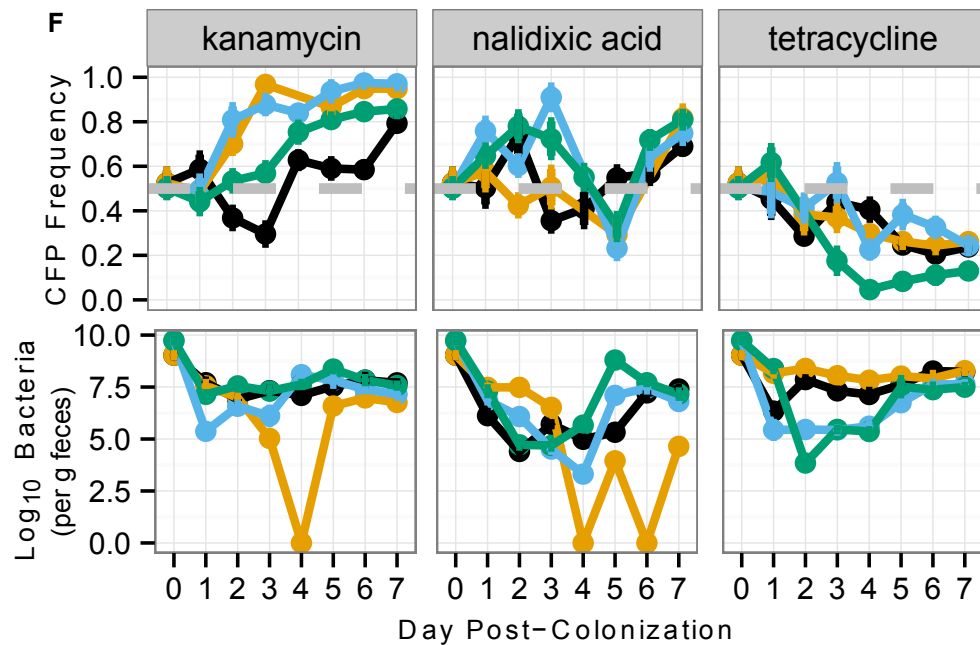

Supplement: Supplementary file 4 [file EVA-9-0994-s004.pdf]

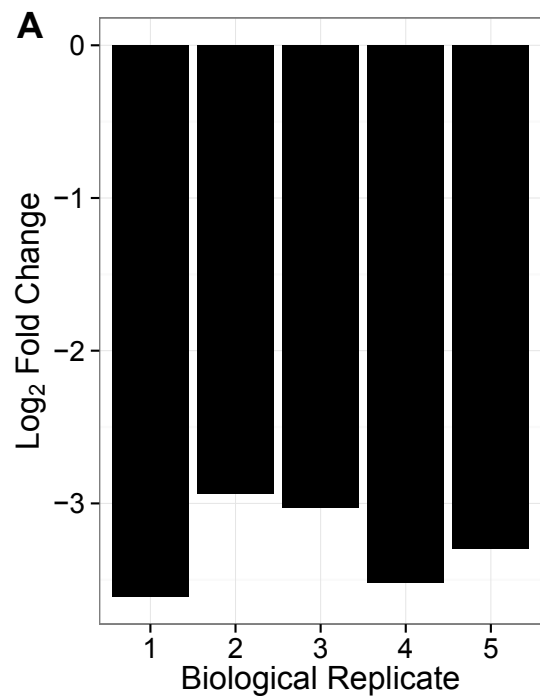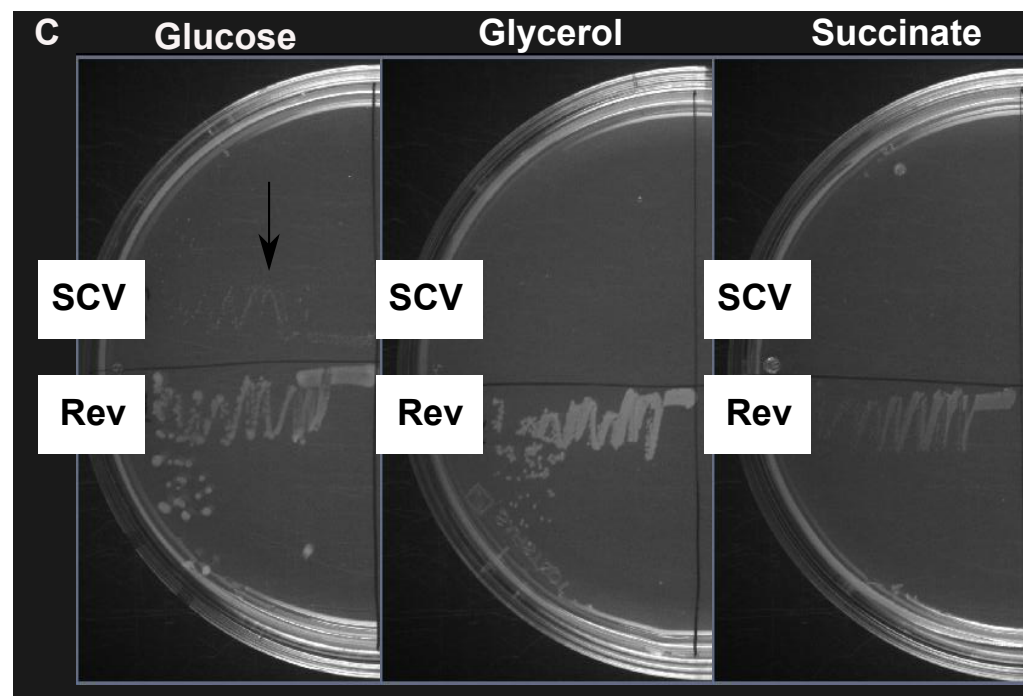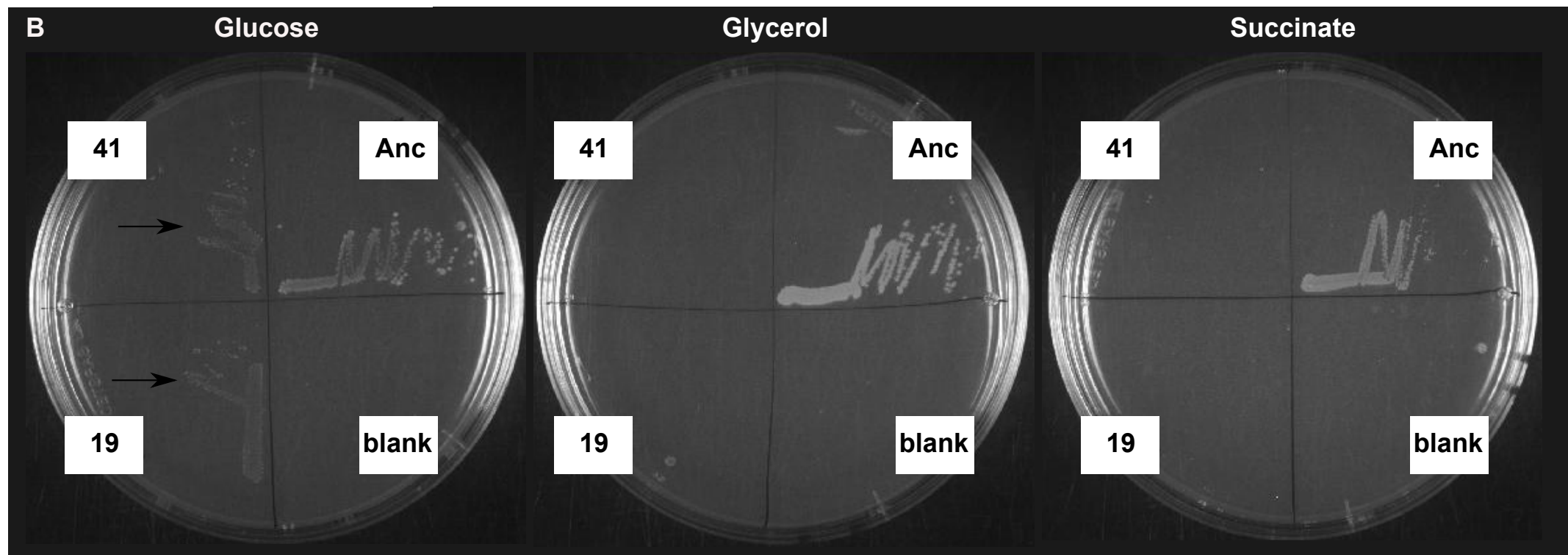

Supplement: Supplementary file 5 [file EVA-9-0994-s005.pdf]

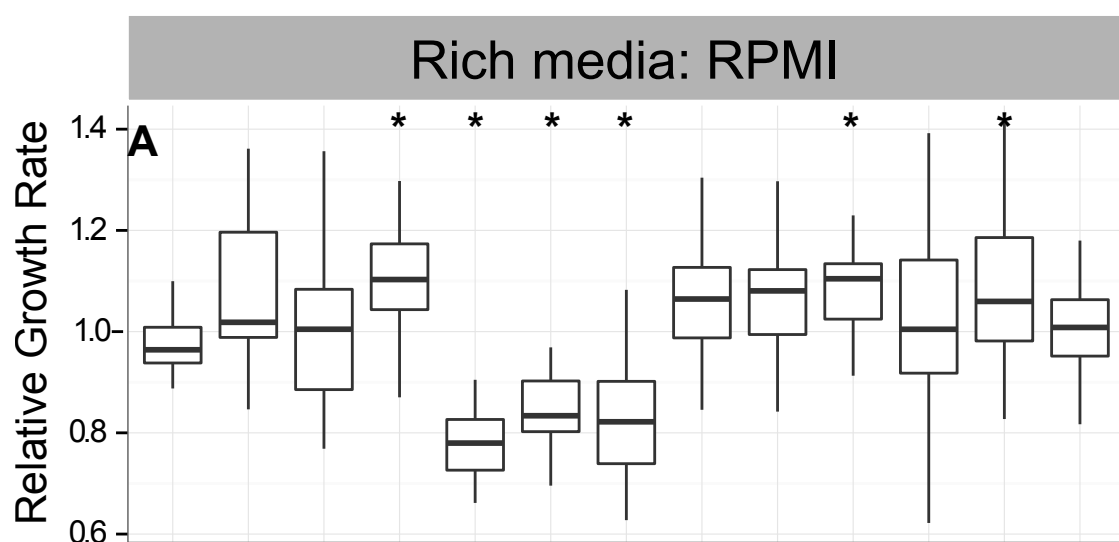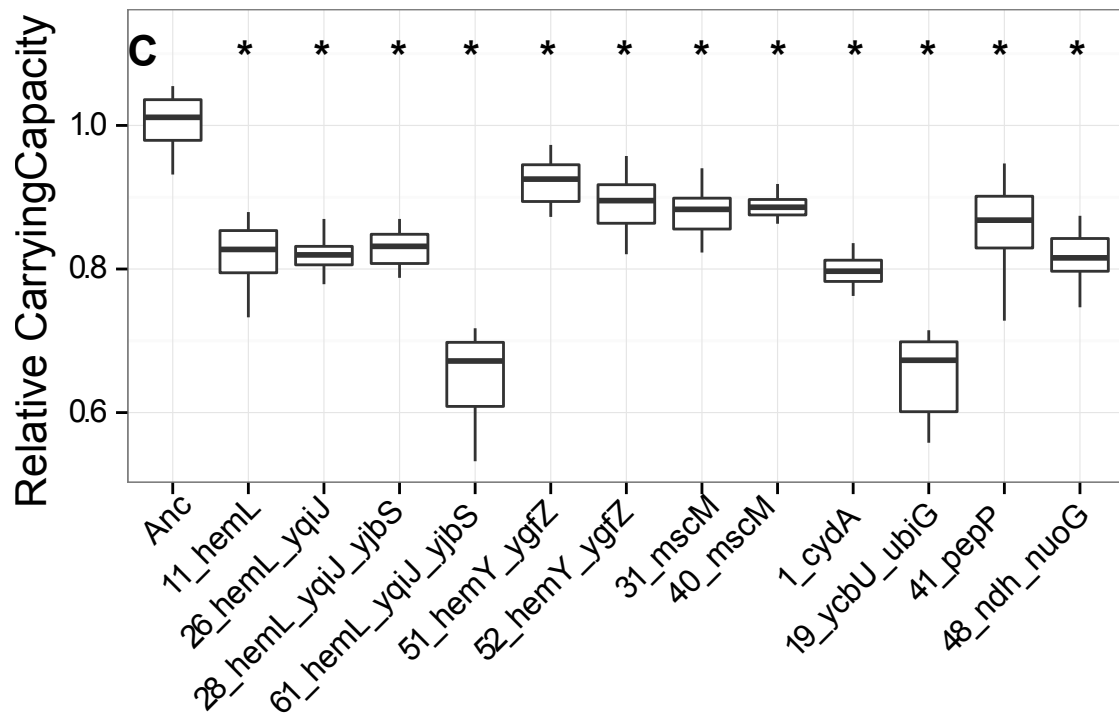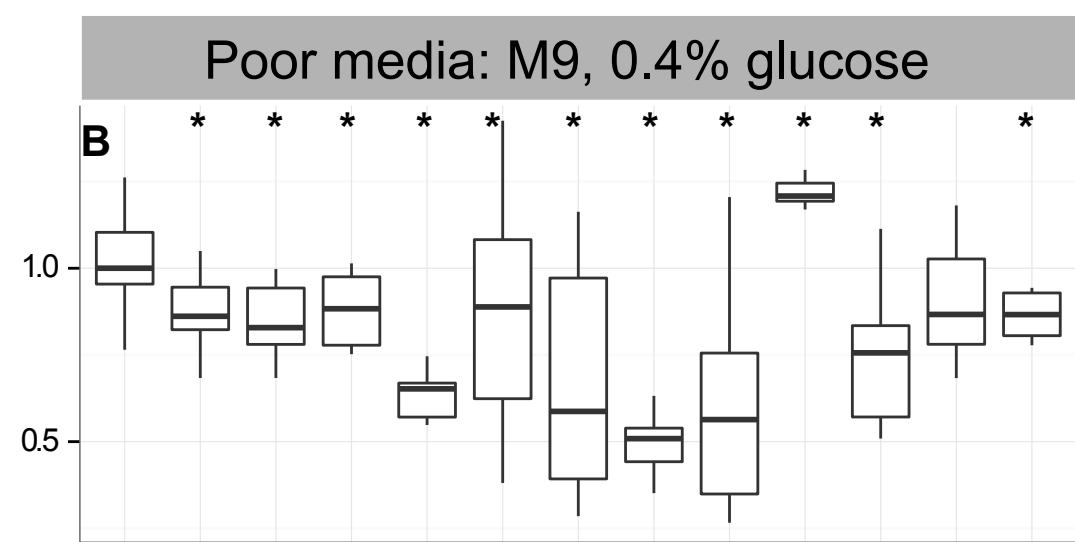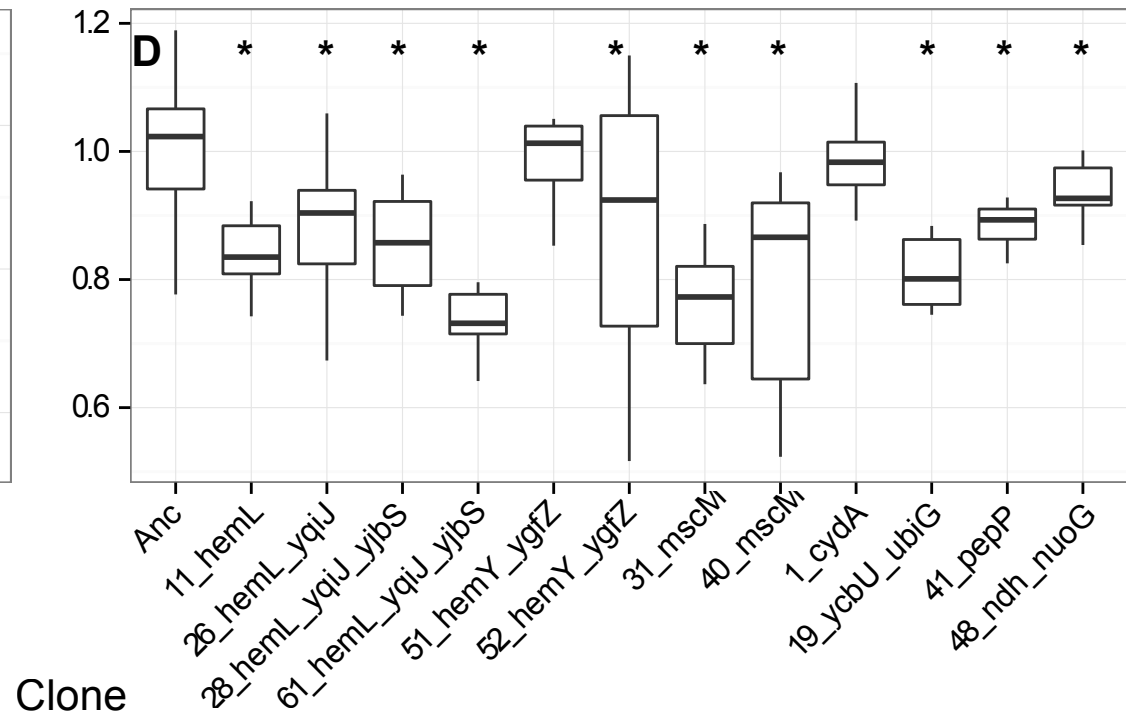

Supplement: Supplementary file 6 [file EVA-9-0994-s006.pdf]

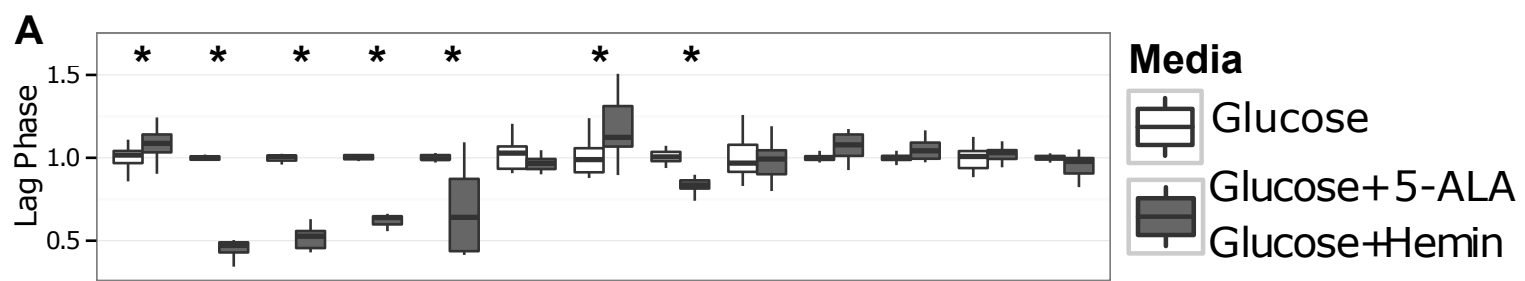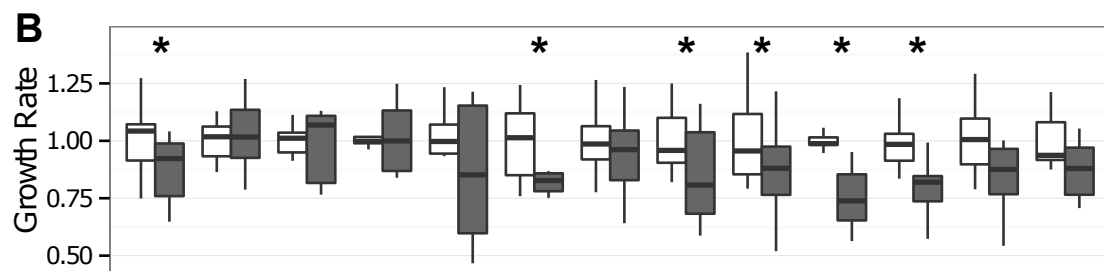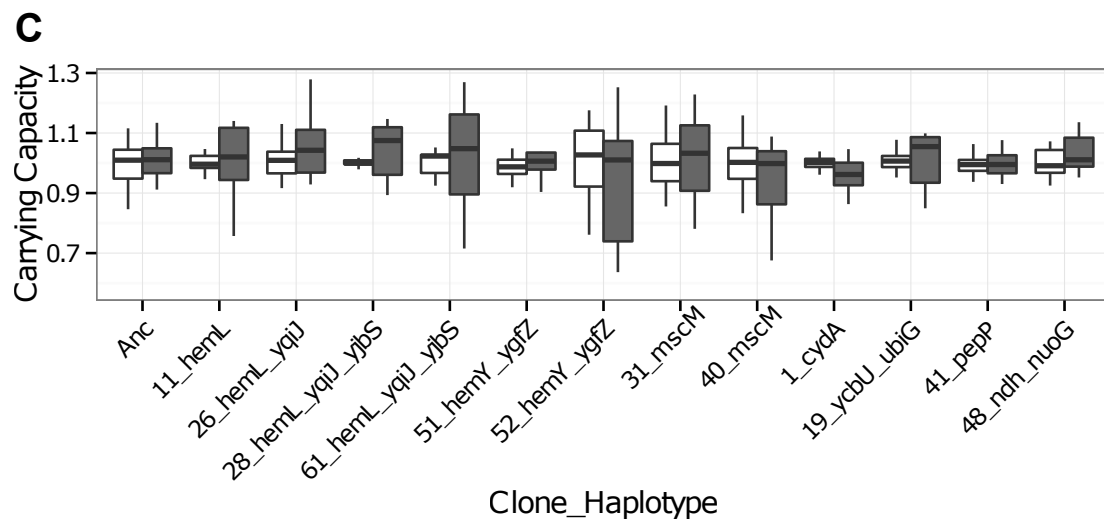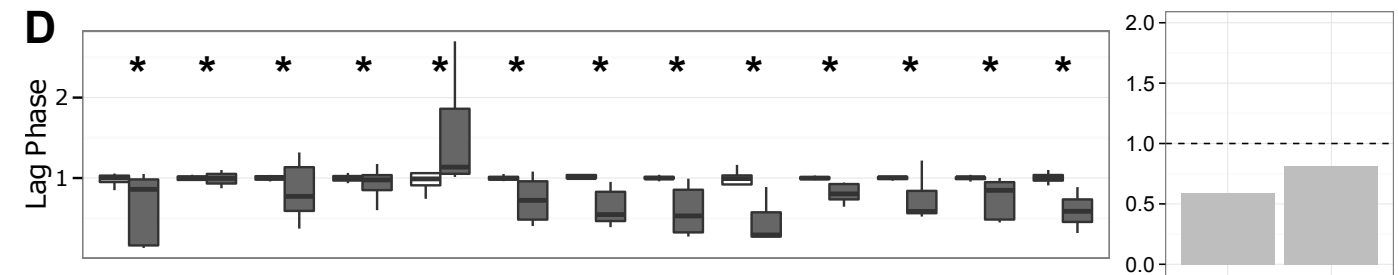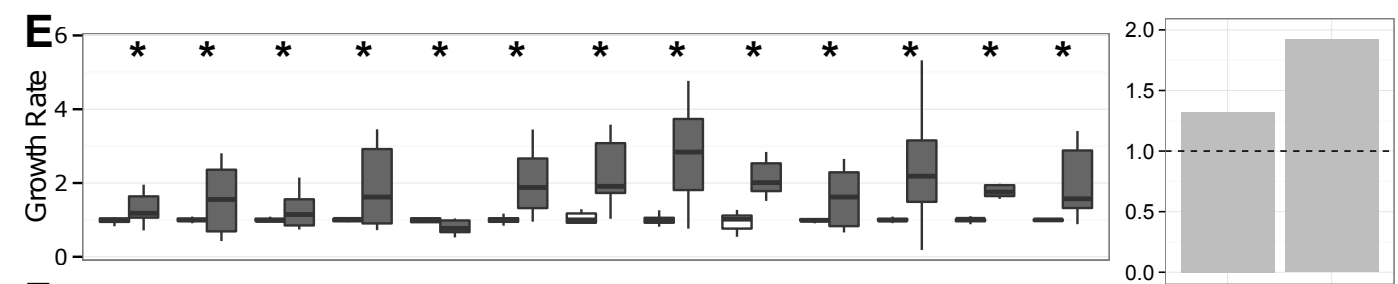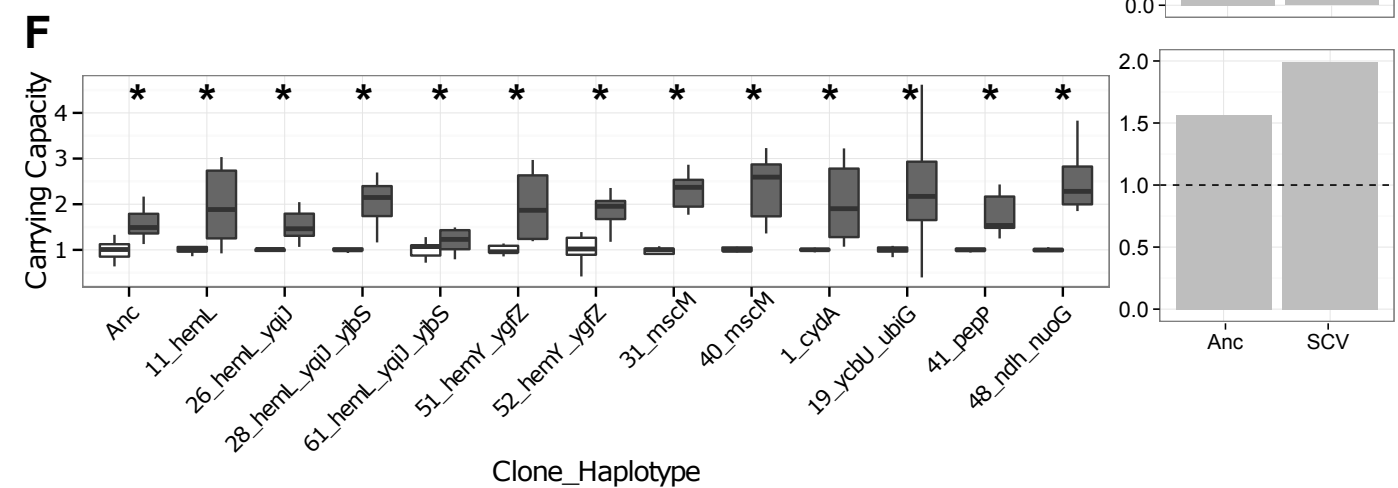

Supplement: Supplementary file 7 [file EVA-9-0994-s007.pdf]

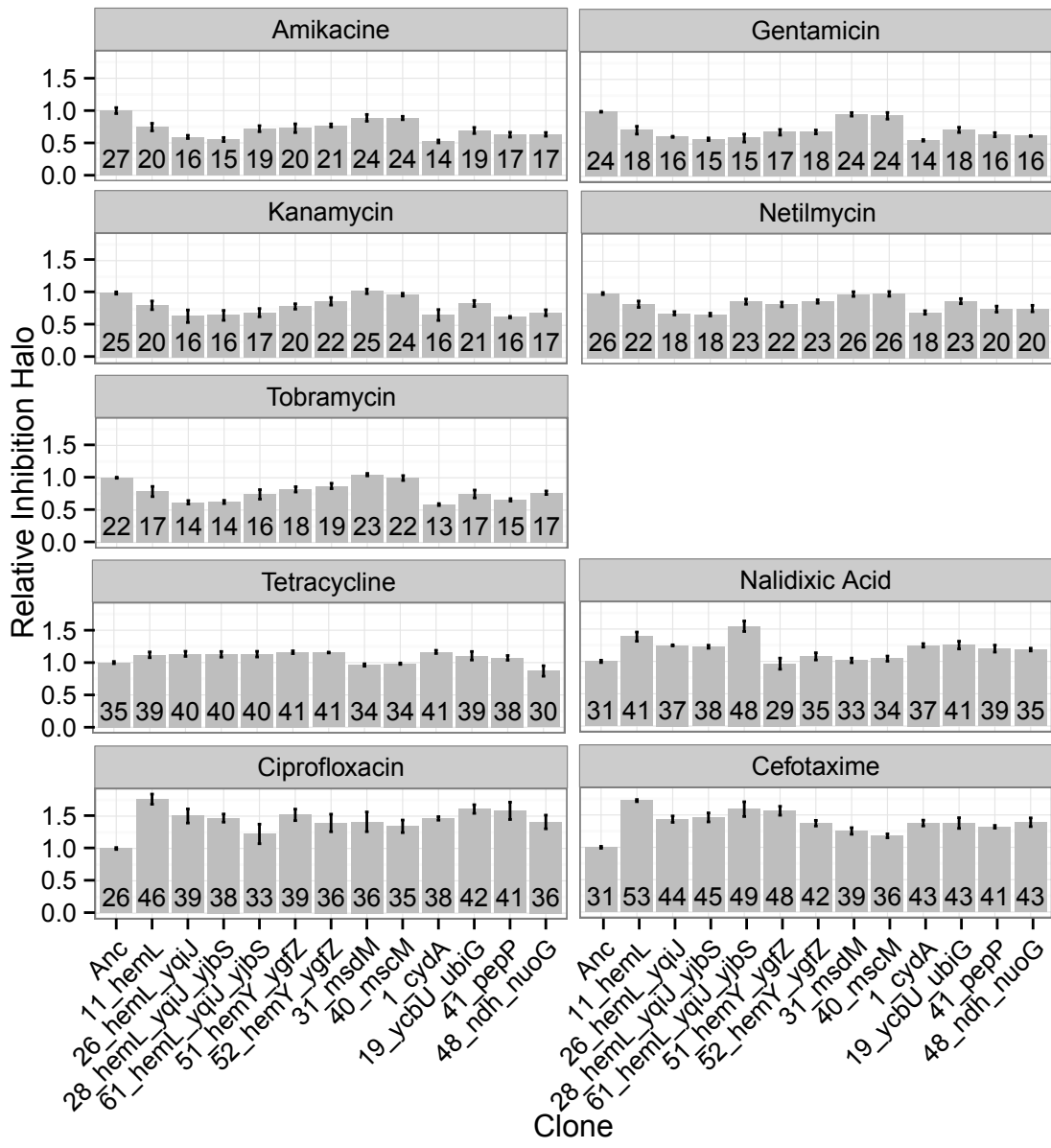

Supplement: Supplementary file 8 [file EVA-9-0994-s008.pdf]
